# Comparing methods for measuring peak look duration: Are individual differences observed on screen-based tasks also found in more ecologically valid contexts?

**DOI:** 10.1016/j.infbeh.2014.04.007

**Published:** 2014-08

**Authors:** Sam V. Wass

**Affiliations:** Medical Research Council Cognition and Brain Sciences Unit, Cambridge, UK

**Keywords:** Infant, Attention, Peak look duration, Sustained attention, Naturalistic attention

## Abstract

Convergent research points to the importance of studying the ontogenesis of sustained attention during the early years of life, but little research hitherto has compared and contrasted different techniques available for measuring sustained attention. Here, we compare methods that have been used to assess one parameter of sustained attention, namely infants’ peak look duration to novel stimuli. Our focus was to assess whether individual differences in peak look duration are stable across different measurement techniques. In a single cohort of 42 typically developing 11-month-old infants we assessed peak look duration using six different measurement paradigms (four screen-based, two naturalistic). Zero-order correlations suggested that individual differences in peak look duration were stable across all four screen-based paradigms, but no correlations were found between peak look durations observed on the screen-based and the naturalistic paradigms. A factor analysis conducted on the dependent variable of peak look duration identified two factors. All four screen-based tasks loaded onto the first factor, but the two naturalistic tasks did not relate, and mapped onto a different factor. Our results question how individual differences observed on screen-based tasks manifest in more ecologically valid contexts.

## Highlights

• Little research hitherto has compared and contrasted different techniques available for measuring sustained attention.
• We took a single cohort of 42 typically developing 11-month-old infants.
• We assessed peak look duration using six different measurement paradigms (four screen-based, two naturalistic).
• A factor analysis suggested that the four screen-based tasks and two naturalistic tasks mapped onto separate factors.
• Our results question how individual differences on screen- based tasks might manifest in more ecologically valid contexts.

## 1 Introduction

Research is increasingly suggesting that early-developing, domain-general aspects of attentional control may mediate subsequent skill acquisition in a variety of areas (e.g. Heckman, 2006; Karmiloff-Smith, 1998; Wass, Scerif, & Johnson, 2012). For example, aspects of domain-general attentional control have been shown to predict, on starting school, children's’ subsequent learning on literacy and numeracy tasks (e.g. Welsh, Nix, Blair, Bierman, & Nelson, 2010). And research into the development of attentional control within clinical disorders suggests that early disruption to attentional control may play a key role in impairing early learning in social settings, for example during word learning, leading to subsequent catastrophic developmental cascades (e.g. Karmiloff-Smith, 1998). This suggests the importance of researching the ontogenesis of attentional control during the first few years of life.

Cohen suggested that infant attention involves at least two different mechanisms: an attention-getting process which determines whether an individual will orient towards a stimulus presented in his periphery, and an attention-holding process which determines how long his attention will be maintained once he fixates (Cohen, 1972). This second phase, the attention-holding process, is commonly described as ‘sustained attention’ (Richards, 2011). However, although individual differences in attention are frequently reported in applied and developmental psychology, the terms used are rarely precisely defined and are conventionally assessed using a variety of methods.

Historically, the most widely used technique for measuring infants’ looking behaviour involves presenting static stimuli using a slide projector or computer screen across a number of discrete but contiguous trials; the infant's viewing behaviour is coded either live by an experimenter viewing the infant on a video feed, or post hoc (Colombo & Mitchell, 2009). Two variables are typically derived: peak look duration, the duration of the longest unbroken look to the screen, and habituation rate, i.e. the rate of change of looks over time. Colombo and Mitchell argued in favour of peak look duration as the better metric of individual and developmental differences in visual attention during infancy because it is more reliable, and shows more robust relationships with long-term cognitive outcomes (Colombo & Mitchell, 1990).

Previous research has demonstrated that peak look duration to novel, static, screen-based stimuli show a U-shaped trajectory over the first year of life (Colombo & Mitchell, 2009; Colombo & Cheatham, 2006; Courage, Reynolds, & Richards, 2006). Research has also robustly demonstrated that peak look duration to novel stimuli during the first year of life relates negatively with long-term cognitive outcomes: shorter look duration during the first year is associated with better performance on later IQ and language measures (Colombo, 1993; McCall & Carriger, 1993; Tami-LeMonda & Bornstein, 1989) and recognition memory (Rose, Feldman, & Jankowski, 2003a, 2003b). Shorter looking is also associated with higher pre-existing knowledge bases and general arousal levels (de Barbaro, Chiba, & Deak, 2011; Dixon & Smith, 2008).

An alternative technique for assessing looking durations during infancy involves presenting dynamic stimuli on a computer screen (Courage et al., 2006; Shaddy & Colombo, 2004; see Richards, 2010 for a review). This work has generally used either TV clips (e.g. Richards & Anderson, 2004) or specially filmed naturalistic or semi-naturalistic dynamic scenes (Wass, Porayska-Pomsta, & Johnson, 2011). These techniques have been used to investigate how autonomic indices change in different attention states (Richards, 2011; Richards & Cronise, 2000), how looking behaviour towards the screen changes over time (Anderson, Choi, & Lorch, 1987; Richards & Anderson, 2004), and how these changes are different in children with Attention Deficit Hyperactivity Disorder (ADHD) (Lorch et al., 2004). To our knowledge, no research has investigated whether individual differences in look duration are consistent across static vs. dynamic looking time paradigms.

A third paradigm that has been used to assess looking durations involves presenting a number of unfamiliar objects consecutively or concurrently in a table-top setting, and performing video coding post hoc to analyse looking behaviour. For example, Kannass and Oakes (2008) videoed 9-month-old and 31-month-old infants playing with toys, in both single-object (objects presented consecutively) and four-object (objects presented concurrently) conditions; they also measured 31-month language performance in the same children (see also Sarid & Breznitz, 1997). They found that shorter look durations in the single-object task correlated with larger vocabularies at 31 months (Kannass & Oakes, 2008). For the multiple object condition, however, they found the opposite relationship: longer durations at 9 months correlated with larger vocabularies at 31 months (see also Choudhury & Gorman, 2000).

**Fig. 1:**
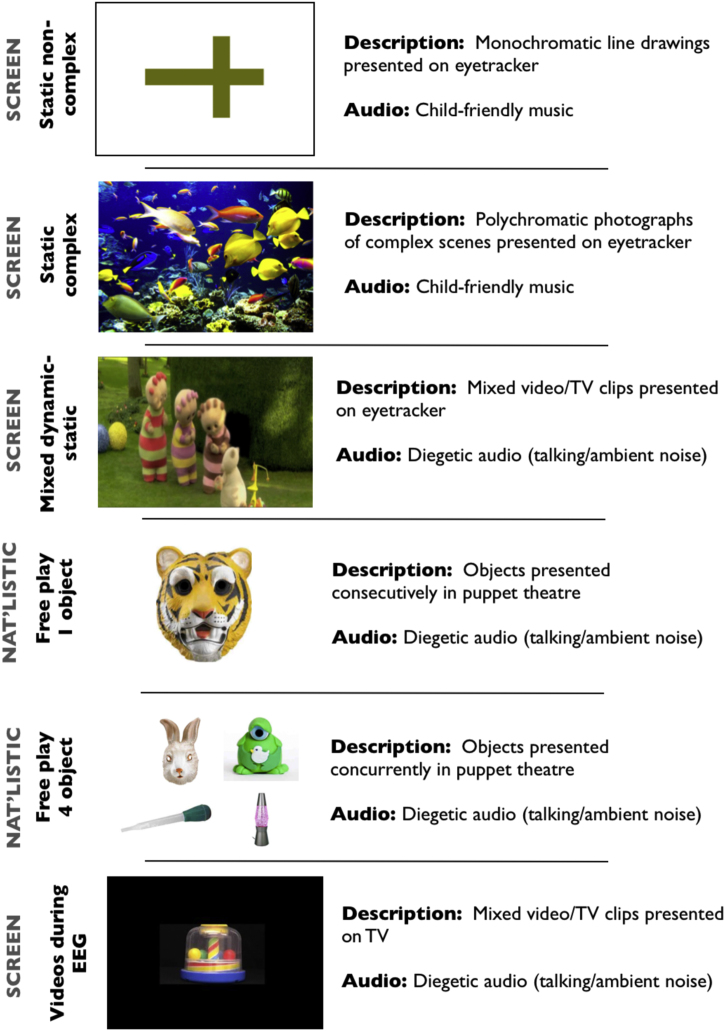
Summary of viewing materials presented.

Despite the strong face similarities between these paradigms, no previous research has assessed whether individual differences using one type of looking time paradigm are consistent across different assessment techniques. A number of studies have addressed this indirectly, but none directly. Kagan and Lewis (1965) examined the relationship between looking behaviour towards static stimuli at 6 and 13 months and the amount of free-play locomotor activity at 13 months, and found that infants with long fixation times at 6 and 13 months were more sedentary during free play. Coldren found that infants’ attention to stimuli in laboratory tasks correlated with the attention to their caregiver in face-to-face interactions at 3- and 4-month-olds but not at 6 months (Coldren, unpublished data, described in Colombo & Mitchell, 1990). Pêcheux and Lécuyer (1983) found with 4-month-olds that fixation time towards static stimuli was positively correlated with their visual exploration of a toy (Fig. 1).

**Fig. 2:**
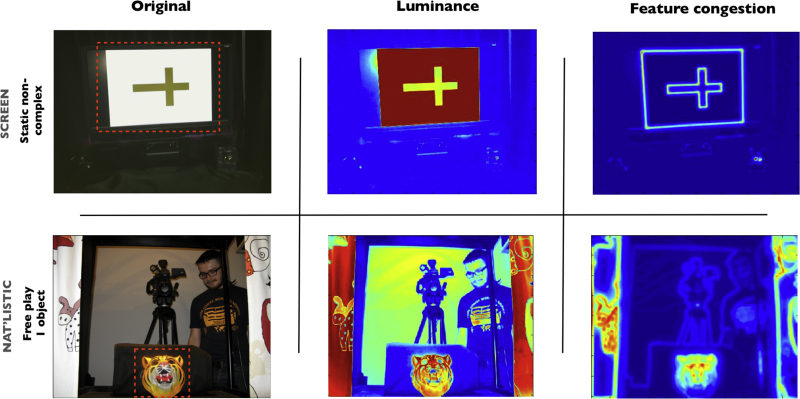
Comparison of paradigms presented from infant's perspective. Top row shows the screen-based looking tasks; bottom row shows the naturalistic looking task. In each row, the left column shows a photo of the stimulus array from the infant's perspective. The active target for the coding of look durations is indicated using a red rectangle. In the central column the luminance of the images is shown. On the right, feature congestion is shown. Feature congestion quantifies local variability across different first-order features such as colour, orientation and luminance (Rosenholtz et al., 2007) and has been shown to influence gaze allocation in contexts in which no motion is present in the field of view (Henderson and Smith, 2009).

This gap in the literature is important for a number of reasons. As we note in Part 2, there are a number of marked differences between these different looking time paradigms, such as: the size of the target towards which attention is being directed, the presence or absence of movement in the target or periphery of the visual field of the child, and the relative luminance of the target relative to other elements within the infants’ field of view (Fig. 2). In the absence of data showing cross-paradigm consistency, we cannot be sure how individual differences in attention as assessed using screen-based tasks might relate to individual differences in attention in naturalistic settings. Are the dissimilarities between screen-based and naturalistic attention tasks documented in Fig. 2 incidental to the individual differences that are assessed on these tasks? Or are they central to them?

Within the habituation literature, shorter looking to static stimuli during the first year is frequently described as an index of ‘faster processing speed’; this is frequently posited as an explanation for the negative correlations noted between look duration during the first year and long-term outcomes (Colombo & Cheatham, 2006). One question that follows from this is: does ‘faster processing’ as assessed using screen-based attention tasks also manifest as different (‘better’, or ‘more efficient’) orienting in naturalistic contexts? Or is shorter looking to screen-based stimuli associated with better long-term outcomes because both measures tap some underlying, ‘pure’ aspect of cognition that is entirely independent of naturalistic orienting? The present study is intended as a small step towards addresing these questions.

### 1.1 The present study

As described above, there exists to our knowledge no previous research that has addressed whether individual differences in peak look duration are consistent across different assessment techniques. The present study was conducted in order to address this question. We presented four screen-based assessments, namely: (i) looking behaviour towards ‘interesting’ (complex) static stimuli, (ii) ‘boring’ (non-complex) static stimuli, (iii) mixed static and dynamic stimuli and (iv) to videos under conditions of distraction (during the recording of EEG data). We also presented two semi-naturalistic looking assessments involving the presentation of novel objects in a table-top setting, in (i) a single-object condition (novel objects presented one by one) and (ii) a four-object condition (four novel objects presented concurrently) (following (Kannass & Oakes, 2008). The six measures were presented in different testing rooms and inter-leaved in order, to a single cohort of typically developing 11-month-old infants. 11-months was chosen as the age for the present study because this has been characterised as an age that shows the first emergence of endogenous attentional control (Colombo & Cheatham, 2006; Courage et al., 2006).

Across all six paradigms, the single dependent variable we assessed was peak look duration. This was selected because it has previously been argued to be the most stable assessment of looking behaviour during infancy–in comparison for example to habituation rate (the rate of change of looks over time), which is less reliable, and shows less robust relationships with long-term cognitive outcomes (Colombo & Mitchell, 1990). As far as possible, peak look duration was assessed identically across the six paradigms we administered.

From reviewing the literature we were able to find no discussions suggesting that different factors might influence peak look duration differentially between screen-based and semi-naturalistic settings. Therefore we predicted that individual differences in peak look duration would be consistent across all the paradigms administered.

## 2 Methods

### 2.1 Participants

42 typically developing 11-month-old infants participated in the study. Mean age at testing was 337 days (range 312–259, standard deviation 9). Gender ratios were 26 male/16 female. Of note, other aspects of these data have already been published elsewhere (Wass et al., 2011; Wass & Smith, 2014). The current data contain, however, completely novel analyses which do not overlap with previous publications.

### 2.2 Apparatus and procedure

The six peak look assessments were administered in three sections. Section A consisted of the ‘static non-complex’ assessment, the ‘static complex’ assessment and the ‘mixed dynamic/static’ assessment. Section B consisted of the ‘structured free play’ assessment. Section C consisted of the ‘videos during EEG’ assessment.

*Presentation order*. All three sections were administered during a single visit, which generally lasted c. 90 min with breaks. Section A was presented in two halves (‘A1’ and ‘A2’). The order in which the sections were administered was: Section A1, then Section B, then Section A2, then Section C. The naturalistic ‘structured free play’ assessment (Section B) was therefore presented between the other screen-based tasks. This design was chosen in order to preclude the possibility of order effects being responsible for the results observed.

*Testing rooms*. Sections A–C were each presented in different rooms. Of note, therefore, the screen-based tasks included in Section C were presented in a different room to the screen-based tasks in Section A.

In the detailed descriptions of the methods that follows, materials are described section by section, together with the data processing techniques that were used.

### 2.2.1 Section A – ‘static non-complex’/‘static complex’/‘mixed dynamic/static’

*Materials*. For the three peak look assessments contained in section 1 infants were seated on their caregiver's lap while the viewing material was presented on a Tobii 1750 eyetracker subtending 24° of visual angle. The three assessments were presented interleaved with each other.

*Static non-complex images*. Two different still images were presented at different stages of the testing protocol. The two ‘non-complex’ images were both monochromatic objects presented against a white background (see Fig. 1 for example). Trials were presented concurrent with child-friendly music, such as songs from *Sesame Street*. Four different songs were used that were paired randomly with the different images. All infants heard the same four songs over the course of all experiments. Trials were presented using a gaze-contingent infant-controlled habituation protocol procedure: images were presented and remained on-screen for as long as the infant looked to the screen. Following cessation of a look, the image was re-presented until two successive looks had taken place that were less than 50% of the longest look so far. In order to confirm eyetracker contact, a small (c. 0.4°) re-fixation target was briefly presented every 15 s; subsequent analyses (described in the Supplementary Materials) suggested that this did not influence the timing of peak look duration measure. Peak look was calculated independently for each image and then averaged.
*Static complex images*. The two ‘complex’ images were polychromatic scenes (see Fig. 1 for example). The testing procedures used were identical to those used for the static non-complex assessment. For practical reasons, individual trials were capped at 120 s; 11 out of the 152 individual trials included reached this cap (see Fig. S1).
To confirm our classification of images into ‘complex’ and ‘non-complex’ feature congestion was calculated for each image using Matlab scripts from Rosenholtz, Li, and Nakano (2007). Feature congestion quantifies local variability across different first-order features such as colour, orientation and luminance; see SM for a more detailed description. For the two ‘non-complex’ images, average feature congestion across the whole frame was found to be 1.7 and 1.6; for the two ‘complex’ images, average feature congestion was 7.6 and 5.1 (see Fig. S2). This confirmed our classification of the stimuli into ‘complex’ and ‘non-complex’.
*Mixed static/dynamic images*. 3 blocks of mixed static and dynamic images were presented at different stages of the testing protocol. Each block lasted 65 s. Each block consisted of a mixture of: head shots of actors (single and in groups) reciting nursery rhymes, still images of actors’ faces, and shots of toys and birds accompanied by background music (see Fig. 1 for example). The individual stimuli within each 65-s block each lasted 4–12 s. As with the static images, a small re-fixation target was briefly presented c. every 15 s in order to confirm eyetracker contact (see analyses in SM).

*Data processing*. Infants’ looking behaviour was coded from a camera on top of the monitor. Gaze was coded in 1-s bins, as either looking at the screen or not. Total percentage looking time and the length of each unbroken look to the target were calculated. Instances in which the participant looked away from and then back to the screen within 1 s were treated as constituting one continuous look rather than two discrete looks. Coder 1 coded 76%, coder 2 48%; 25% of the videos were double coded. Cohen's *kappa* was calculated to assess inter-rater reliability and was found to be 0.71.

### 2.2.2 Section B – ‘structured free play’

*Materials*. The two peak look assessments contained in Section 2 were conducted in a puppet theatre with attractive surrounds, and a stage behind which experimenter and the camera were visible. Infants sat on their caregiver's lap, close enough to the stage so that they could reach to and touch the objects on it. Between each trial, the curtains of the puppet theatre were closed and new objects were placed on the stage; reopening them marked the start of the next trial.

The two assessments were presented consecutively:

*Free play – 1-object condition*. In the single-object condition, five objects (an plastic figure/a basting pipette/a glitter lamp/a lion mask/a rabbit mask) were presented in randomised order consecutively for 30 s each. Fig. 1 shows an example of the objects used; Fig. S3 shows images of all the objects used. The objects used varied in size from 5–20 cm.
*Free play – 4-object condition.* The four-object condition was presented immediately after the one-object condition. Four objects (a rubber duck, a plastic train, a plastic teddy bear, a tiger finger puppet) were presented concurrently in a line across the stage, in a randomised order, for 90 s. The objects used varied in size from 5 to 10 cm. Data from 10 participants was unusable for the four-object condition due to changes made to the experimental protocol during testing.

*Data processing*. Infants’ looking behaviour was recorded from a camera positioned behind the stage. The coding protocol used was based on that used by Kannass and Oakes (2008). Infants’ looking behaviour was coded for whether the infant was looking at the object or not. Sections where the object was not on the stage (because the infant had knocked or thrown it off) were excluded. All coding was conducted in 1-s bins. Data were triple coded. Coder 1 coded 70%, coder 2 50% and coder 3 24%; 40% were double coded by coders 1 and 2 and 24% by coders 1 and 3. Cohen's *kappa* was calculated to assess inter-rater agreement. This was found to be 0.72 between coders 1 and 2 and 0.78 between coders 1 and 3.

### 2.2.3 Section C – ‘videos during EEG’

*Materials*. The peak look assessment contained in Section 3 was presented with infants sitting on their caregiver's lap while viewing materials were presented on a cathode ray TV subtending 30° of visual angle. Simultaneously with the administration of this task, infants were having EEG data recorded using a 128-channel EGI hydrocel net (Wass, 2011).

Only one assessment was presented in this section:

*Videos during EEG*. Three videos were presented sequentially in rotation during EEG recording. These videos were: (i) a series of actresses reciting nursery rhymes to camera; (ii) videos of toys spinning; (iii) a short TV clip. Videos lasted 32–44 s each. Each video was presented twice.

*Data processing*. Infants’ looking behaviour was recorded from a camera positioned below the monitor. Videos were coded according to whether the infant was looking to or away from the screen, using an identical coding scheme to that used in sections and 2. Coder 1 coded 76%, coder 2 48%; 24% were double coded. Cohen's *kappa* was calculated to assess inter-rater reliability and was found to be 0.88.

## 3 Results

The results section is in two parts. Firstly, descriptive statistics of the looking time data obtained from the different paradigms are presented. Secondly, analyses are presented that examine the inter-relationships in looking time across the different assessments administered. Specifically we wished to evaluate the hypothesis that individual differences in looking time would be consistent across the six assessments.

### 3.1 Part 1 – Descriptive statistics of looking time data

Descriptive statistics for the entire data set are shown in Table 1. For each assessment, mean peak look oberved across all infants has been reported, together with the Standard Error of the Mean (S.E.M.) and range. Additionally, for comparison, identical data have been reported for mean look duration (i.e. the average of all looks recorded towards the stimuli). For each assessment, the number of participants who provided usable data is shown in the final column. With the exception of the free-play 4 object task, for which (as described above) changes were made to the experimental protocol during testing, drop-out rates are acceptable (maximum 4/42). These were due to fussiness and non-compliance during testing.

**Fig. 3:**
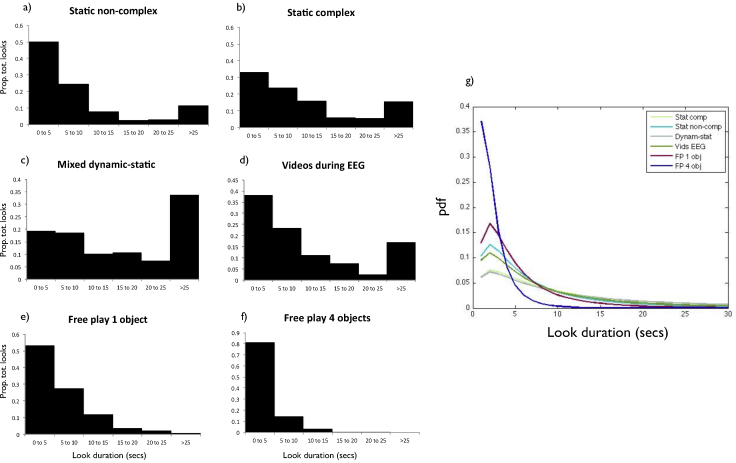
Histograms and log normal distribution fittings. Panels (a)–(f) show histograms of all peak looks observed across the different assessments we administered. Panel (g) shows lognormal distributions of peak looks observed.

Fig. 3a–f shows histograms of all the individual looks collected on the different tasks. Fig. 3g shows plotted lognormal fittings. Lognormal distributions were calculated as these are generally reported to be the best fit on infant looking time data (Pempek et al., 2010; Richards & Anderson, 2004). Marked differences in the patterns of look durations observed on different tasks can be seen: both peak and mean look duration were higher for all of the screen-based tasks than for the structured free play tasks. Within the screen-based tasks, markedly longer peak looking times were observed in the static complex and mixed dynamic-static categories than in the other categories.

The between-participant distributions of peak look durations were found to be positively skewed, in common with all looking time assessments (see e.g. Richards & Anderson, 2004); therefore all subsequent analyses have been calculated based on log-transformed data (following e.g. Frick, Colombo, & Saxon, 1999).

### 3.2 Part 2 – Analyses to examine the inter-relationships in looking time across the different assessments administered

We wished to evaluate the hypothesis that individual differences in looking time would be consistent across the six assessments we administered. In order to examine this, two analyses were conducted. First, zero-order correlations were calculated. Second, a factor analysis was performed.

**Fig. 4:**
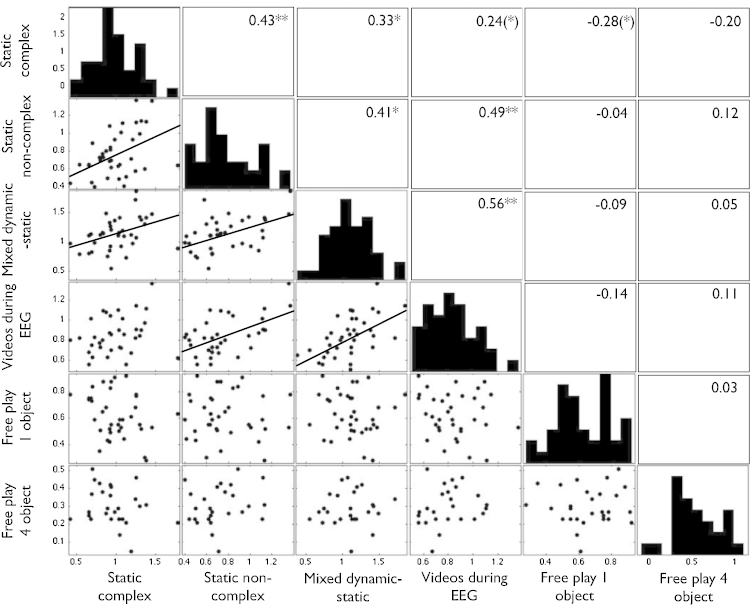
Correlation matrix showing the relationship between the dependent variables entered into the factor analyses. Histograms showing the distribution of each variable following a log transform are shown diagonally on the 1:1 line. Below this line, scatterplots show the relationships between variables. For those variables showing a bivariate correlation at *p*(2-tailed) > .05, a linear regression line has been drawn in black. Above the 1:1 line, the Pearson's product moment correlation shows the relationship between the two variables. The stars show the significance levels of the bivariate correlation: **p(2-tailed) < .01, *p < .05, and (*)*p* < .10.

First, histograms and scatterplots were calculated to assess whether per-participant peak look values derived from the log-transformed data were parametrically distributed, and whether any bivariate relationships observed were robust. Fig. 4 shows these scatterplots. All parameters were found to be normally distributed.

Zero-order correlations. Fig. 4 shows the zero-order bivariate correlations that were observed between the variables entered into the factor analysis. The four screen-based tasks (static complex, static non-complex, mixed dynamic-static, videos during EEG) all show significant correlations (*r* = .33 to .56, all *p*s < 05) with the exception of the static complex to dynamic during EEG (*r* = .24, *p* < .10). Inspection of the scatterplot (Fig. 4) suggests that this relationship is weakened by an outlier. In comparison the two FP tasks do not correlate with any of the screen-based tasks (negative in 5 of the 9 comparisons conducted, and *r* < = .12 in the remaining 4). The scatterplots in Fig. 4 suggest that this not attributable to the presence of outliers.

One explanation that was considered for the low zero-order correlations observed with the free play data was that these data were inherently more ‘noisy’ than the screen-based looking time data. In order to evaluate this possibility, data were examined from an overlapping dataset that has been published previously (Wass, 2011; Wass et al., 2011). In this paper, an identical task to that presented here was presented twice at fifteen days’ interval to a smaller cohort (*N* = 21) of infants. Analyses assessed the number of total attentional reorientations and attentional shifts from object to person. Test-retest reliability between the two testing sessions was *r* = .53, *p* < .01 for total attentional reorientations, and *r* = .52, *p* < .05 for attentional shifts from object to person. This suggests that these measures are relatively stable as indices of individual differences.

Factor analysis. Factor analyses were conducted to examine the factorial structure underlying our data in more detail. Our analytical approach was based on that used in previously published research (Rose, Feldman, & Jankowski, 2004, 2005). First, the sample size was examined. The ratio of participants to variables for the factor analysis was found to be 6.3, which is above the prescribed ratio of 5 suggested by Hair, Tatham, Anderson, and Black (1998). To maximise the sample size for factor analysis, missing values were imputed based on the mean of the subscale to which that item belonged (following Blair & Razza, 2007).

The factor analysis yielded a two-factor solution (Eigenvalues >1.0) representing 59% of the total variance (see Table 2). These two factors were submitted to a principal axis rotation (oblimin) and the scree plot was inspected, supporting a two-factor solution. Thresholds were set at 0.70 for principal loading and 0.50 for secondary loading (Guadagnoli & Velicer, 1988).

The first factor, which had an Eigenvalue of 2.29 and accounted for 38% of the variance, was defined by three of the screen-based tasks, with the fourth screen-based task allotted a secondary loading. The second factor, with an Eigenvalue of 1.23, was loaded onto by the two FP variables (4-object as primary loading and 1-object as secondary loading), and (negatively) by the static complex variable (primary loading).

## 4 Discussion

Our analyses were designed to assess whether individual differences in peak look duration are consistent across different looking time measurement paradigms. To 42 typically developing 11-month-old infants we administered six assessments of peak look duration, including four screen-based assessments and two free-play based assessments. We predicted that results obtained would be consistent across all paradigms. The results were not as predicted. The factor analysis suggested a two-factor solution. The first factor was defined by the four screen-based tasks (‘static non-complex’, ‘mixed dynamic-static’ and ‘videos during EEG’, with ‘static complex images’ as a secondary loading). The second factor was defined by the two free play tasks (one as a secondary loading) and also (with a negative loading) by the static complex screen task.

The four screen-based tasks were administered across different testing rooms, and interspersed with the free play tasks, which precludes the possibility of room or order effects being responsible for our results. Looking time to static screen stimuli and to dynamic screen stimuli showed strong correlations. Strikingly, we also found that looking behaviour towards a TV screen during recording of EEG data, which has the additional variance of tightness of fit of the EEG net, reactivity to testing and so on, mapped onto the same factor as the other three screen-based tasks, that were administered using a different screen in a different room. In contrast the two FP assessments mapped onto a separate factor, and showed non-significant (max *r* = .12) zero-order correlations with each of the screen-based tasks. The zero-order correlations observed between the FP and screen-based tasks were negative in 5 out of 8 comparisons. In the factor analysis, the only screen-based task (static complex) that loaded on to the second factor loaded on *negatively* (higher looking time to static complex images associated with lower looking time during structured free play). Further analyses were conducted to assess the possibility that these findings might be attributable to other factors such as increased measurement error during the administration of the free play tasks, with negative results.

There are a number of limitations to this study. The sample size was relatively small (*N* = 42), and a number of techniques used by other researchers to complement looking time measures (such as heart rate measurement and focused attention coding) were not applied. Furthermore, measurements were only taken with one age group (11-month-olds), whereas the limited data available suggests that different results may have been observed if the experiment were repeated with younger infants (Coldren, unpublished data; discussed in Colombo & Mitchell, 1990).

Nevertheless, our results suggest that, in 11-month-old infants, individual differences in peak look duration are constant across different screen-based tasks but not between screen-based and semi-naturalistic tasks. We were able to find no discussion in the literature suggesting that different factors might influence peak look duration between screen-based and semi-naturalistic settings. What kinds of differences might these be? The following discussion is structured around a number of factors commonly thought to influence peak look duration.

The first factor commonly associated with peak look duration is processing speed. Sokolov argued that the initial presentation of a novel stimulus produces a conflict between a “neural model” of the current environment and the sensory processes occurring in the brain; prolonged exposure to that stimulus allows the viewer to form an internal representation of it, which is why looking durations decline over time (Sokolov, 1963). ‘Faster processors’ are thought to require less time to form an internal representation; this is frequently linked to the finding that shorter peak look duration to static stimuli during the first year correlates negatively with long-term cognitive outcomes (e.g. Rose et al., 2002; Rose, Feldman, Jankowski, & Van Rossem, 2008).

Advocates of the importance of processing speed in influencing peak look duration might predict that individual differences would not be stable between looking time to static screen and dynamic screen stimuli, since one involves static visual information and the other constantly changing information. They might also suggest that individual differences might be stable between static screen and our free play task, since both require forming internal representations of static targets (on-screen pictures and ‘real-world’ objects). In fact we found the opposite pattern: individual differences in peak look duration were consistent across the static screen and dynamic screen stimuli but not with the free play task.

A second factor related to peak look duration is ease of disengaging of visual attention. Frick and colleagues measured the relationship between experimentally assessed attentional disengagement latencies and spontaneous looking behaviour to static screen stimuli in typically developing 3- and 4-m-os (Frick et al., 1999). They found that long-looking infants showed greater variability in their response latencies. This suggests that, at least in younger infants than the 11-month-olds studied here, attentional disengagement may play a role in mediating spontaneous looking behaviour.

This is one area where differences can be noted between our screen-based and semi-naturalistic paradigms (see Fig. 2). Screen-based paradigms tend to be designed with the screen occupying a relatively large proportion of the infant's visual field (typically c.25° of visual angle, as here), whereas in FP paradigms the target is generally much smaller (c. 5° in our case). In screen-based tasks the target is generally much more luminant than the surrounds (which are typically dark); in our free play paradigms, in contrast, this was not the case (see Fig. 2). Lastly, in screen-based tasks there are sharp luminance contrasts between the edge of the screen and the surrounds; again, these were not present in the FP task (see Fig. 2). These differences may be important because previous research has noted that viewers tend to dwell on areas of high luminance contrasts such as object boundaries. Although this effect has been reported at all ages from 6-week-old infants (Bronson, 1994) through to adults (Henderson & Smith, 2009) its effect has been reported to decline with increasing age (Frank, Vul, & Johnson, 2009; Karatekin, 2007). The high-contrast and prominent luminance contrasts present in our screen-based but not in our naturalistic tasks may influence behaviour in the current study, and perhaps more for some infants than others.

A third factor that may relate to peak look duration is autonomic arousal. This can be assessed in both phasic (i.e. event-related) and tonic contexts (de Barbaro et al., 2011; Richards, 2011). Richards and colleagues explored changes in heart rate variability and peak look during object examination; they found a decrease in variability during attention, which was interpreted as consistent with a model of phasic parasympathetic vagal influence on the heart during sustained attention phases (Richards & Casey, 1991; Richards, 2011). Of note, our screen-based tasks (particularly the static screen stimuli) contained abrupt changes in luminance coincident with the onset of each trial: the screen transitioned from dark to bright in an otherwise darkened room and an auditory stimulus was presented; such changes were completely absent in the naturalistic task. It is possible that these abrupt changes in luminance are associated with phasic changes in sympathetic/parasympathetic nervous system balances, and that some infants are more susceptible to these changes than others (Alkon et al., 2006). This factor would influence looking behaviour in the screen-based but not the naturalistic looking time tasks.

Aston-Jones and colleagues suggested a role for brainstem, Norepinephrine modulated arousal systems in shifting between attention states; they distinguish between a ‘scanning’ mode, in which look durations are short and the focus of visual attentiveness is wide, and a ‘focused’ mode, in which look durations are longer and the spatial distribution of attention is narrower (Aston-Jones, Rajkowski, & Cohen, 1999; Aston-Jones, Iba, Clayton, Rajkowski, & Cohen, 2007; cf. Pannasch et al., 2008). In our semi-naturalistic tasks, other targets (objects and people) are present within the peripheral visual field of the infant, whereas attempts were made to ‘black out’ all peripheral objects for the screen-based tasks (as is typical in other labs) (see Fig. 2). The shifting between attention states that Aston-Jones and colleagues describe may therefore be a factor in our semi-naturalistic tasks but not in our screen-based tasks.

A fourth factor that may relate to peak look duration is executive control. Aspects of executive control have been reliably associated with sustained attention in older children (e.g. Reck & Hund, 2011). (Note however that sustained attention in these studies with children is assessed not using looking time measures but with tasks such as the Continuous Performance Task, whose relationship with peak look duration has, to our knowledge, not been studied.) Colombo and Cheatham point out that positive correlations are observed between long-term cognitive outcomes and peak look to static stimuli after the first year of life, whereas negative correlations are observed between the same two variables during the first year. They suggest that this may be attributable to the emergence of effortful control as a factor mediating behaviour at about the 12-month boundary (Colombo & Cheatham, 2006; see also Courage et al., 2006). Note, however, that Kochanska and Aksan (2006) found that focused attention (not peak look duration) during the second half of the first year correlated *positively* with effortful control at 22 months.

One difference between our screen-based and our semi-naturalistic tasks may be relevant here. This is that, for the semi-naturalistic tasks, a number of other informative gaze targets (such as the experimenter) are within the field of view of the child – whereas the screen-based tasks were conducted in a darkened room. There are a variety of reasons why looks away from the object may have an adaptive value in our free play paradigm but not in our screen-based paradigm (e.g. Rueda, Posner, & Rothbart, 2005; Sheese, Rothbart, Posner, White, & Fraundorf, 2008). It may be therefore that executive control relates more strongly to peak look duration in the free play than in the screen-based tasks, although future work is required to investigate this in more detail (cf. Rothbart, Ellis, Rueda, & Posner, 2003; Sheese et al., 2008).

## 5 Conclusions

To a cohort of typically developing 11-month-old infants we presented several assessments of peak look duration, including some that assessed looking behaviour on screen-based tasks and others that assessed behaviour on semi-naturalistic tasks. We found that the four screen-based tasks (looking to static non-complex, to static complex, to mixed dynamic/static and to dynamic stimuli during EEG recording) all mapped onto a single factor, whereas the two free play assessments mapped onto a separate factor. In our discussion we noted a number of ways in which factors such as susceptibility to high luminance contrasts and abrupt stimulus onset–offset changes may be key factors mediating individual differences on screen-based tasks, but relatively unimportant in more naturalistic contexts. Future research should exploit recent technological advances such as head-mounted eyetrackers (e.g. Aslin, 2009) to increase our understanding of how individual differences in naturalistic attention relate to individual differences in infant attention as assessed using screen-based paradigms.

## Figures and Tables

**Table 1:** Descriptive statistics.

		Peak look duration(s)	Mean look duration(s)	*N*
*Screen*				
Static complex	Mean (StErr)	63.3 *(42.1)*	21.5 *(15.3)*	39
	Range	6.5–120.3	3.8–76.9	
Static non-complex	Mean (StErr)	29.3 *(23.6)*	10.9 *(8.5)*	39
	Range	5.5–90.2	2.8–35.4	
Mixed dynamic-static	Mean (StErr)	57.6 *(18.5)*	23.5 *(13.7)*	40
	Range	16.0–90.0	5.1–72.3	
Videos during EEG	Mean (StErr)	*40 (11.8)*	12.1 *(5.4)*	38
	Range	17.0–84.0	5.7–30.0	

*Structured free play*				
Free play 1 object	Mean (StErr)	*18.0 (6.8)*	6.2 *(2.6)*	38
	Range	6.0–30.0	2.3–12.7	
Free play 4 objects	Mean (StErr)	9.5 *(3.7)*	2.7 *(0.9)*	28
	Range	3.0–17.0	1.2–5.5	

**Table 2:** Two-factor solution. Bold indicates principal loading. Italicised bold indicates secondary loading.

	Factor 1	Factor 2
Static complex	***0.51***	**−0.71**
Static non-complex	**0.79**	−0.11
Mixed dynamic-static	**0.78**	−0.13
Videos during EEG	**0.81**	−0.04
Free play 1 object	−0.17	***0.58***
Free play 4 object	0.24	**0.70**
Eigenvalues	2.29	1.23
% of variance	38.2	20.4
Cumulative %	38.2	58.6
