# Supplementary material for: Comparing methods for measuring peak look duration: Are individual differences observed on screen-based tasks also found in more ecologically valid contexts?
Source: Infant Behav Dev. 2014 Aug;37(3):315–25. doi: 10.1016/j.infbeh.2014.04.007 (PMC4103485; doi:10.1016/j.infbeh.2014.04.007)
Supplement: Supplementary file 1 [file mmc1.pdf]

Supplementary materials

Supplementary methods

Section 1 – static non-complex, static complex and mixed static/dynamic assessments

In order to confirm eyetracker contact, a small re-fixation target subtending c. 0.4˚ of visual angle was briefly presented every 15 seconds. In order to assess the possibility that the presence of this re-fixation stimulus might have influenced our results we plotted a cumulative frequency distribution of the peak look duration of all of the individual trials (four per infant - two complex, two non-complex) that were recorded. We reasoned that, if the presence of this target had influenced the peak look duration measure, then peaks would be observable in this frequency distribution immediately after the target was presented (i.e. at 15, 30, 45 seconds and so on). Indeed, small peaks are detectable at these times, consistent with this hypothesis. However, it can also be seen that these peaks are small relative to the range of response times observed. We concluded therefore that there was no evidence to suggest that the presence of this re-fixation target invalidates our use of this measure as an assessment of peak look duration to static images.

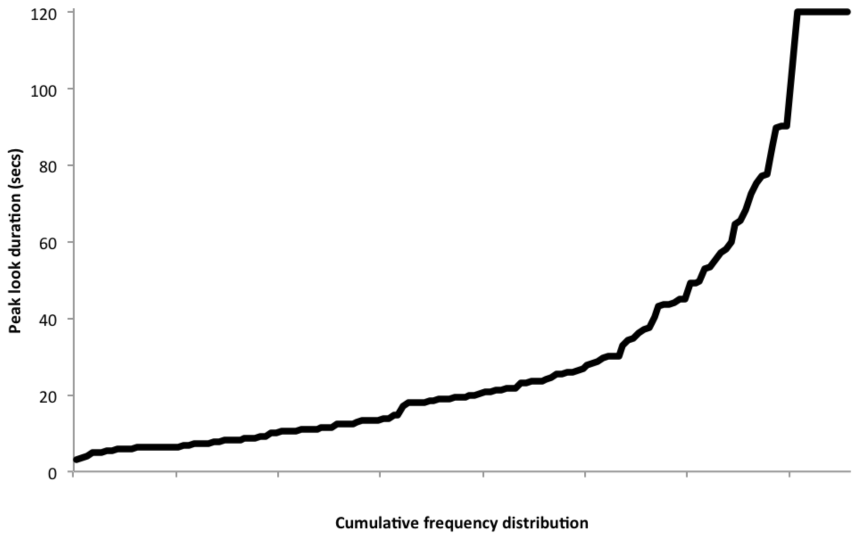

Figure S1: Cumulative frequency distribution of all trials presented during the assessment of looking time to static images.

Feature congestion calculations

Feature congestion was calculated using Matlab scripts that are described in detail by Rosenholz and colleagues (Rosenholtz et al. 2007). These were chosen in preference to the more widely used salience models (e.g. Itti & Koch, 2001) since these models make a lot of assumptions about how attention is controlled, such as the inclusion of inhibition of return and winner takes all in the salience computation. Briefly, the input image was converted into the CIELAB color space and then processed at three scales by creating a Gaussian pyramid by alternately smoothing and subsampling the image (Burt & Adelson, 1983). Features were then identified based on luminance contrasts (by filtering the luminance band by a center-surround filter formed from the difference of two Gaussians and squaring the outputs); color, performed at each scale by pooling with a Gaussian filter; and orientation (using a two-vector, (k cos(2θ), k sin(2θ)), at each image location and scale, where θ is the local orientation and k is related to the extent to which there is a single strong

orientation at the given scale and location. Then the local (co)variance for each feature is calculated; these are then combined, scaling the clutter value in each feature dimension by the range of possible clutter values for that feature (Rosenholtz et al., 2007).

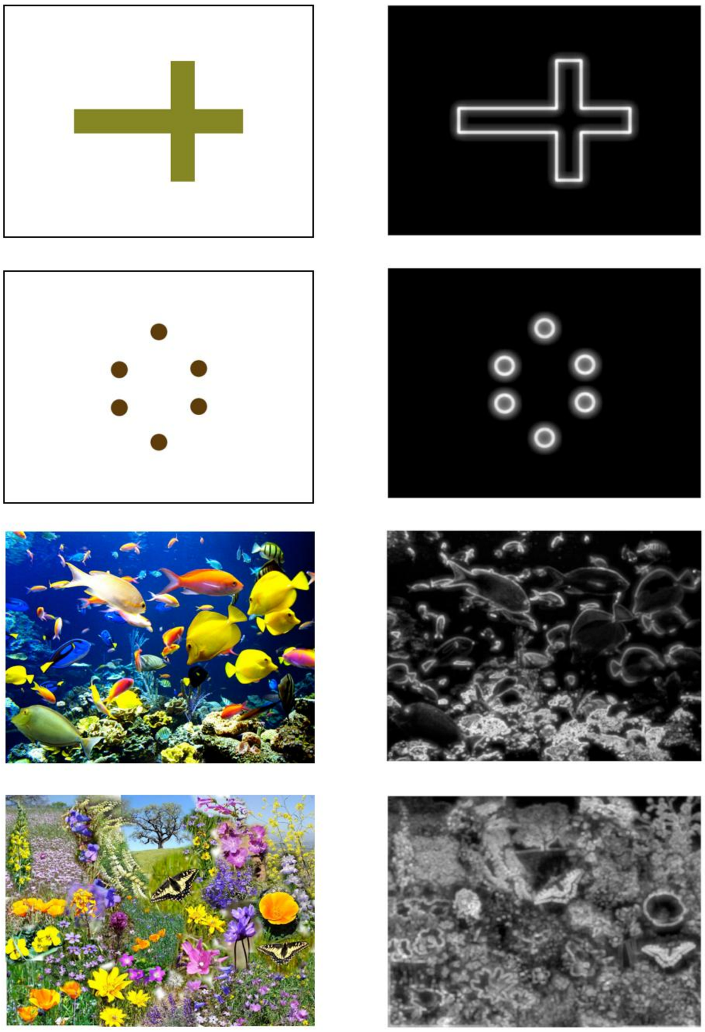

Figure S2: Feature congestion calculations used to quantify simulus complexity. The images on the left show the original image. From top to bottom, the top two images were selected as 'non-complex' and the bottom two images were selected as 'complex'. The images on the right are those outputted by scripts from Rosenholz and colleages. Areas drawn white are areas with relatively high feature congestion, defined via differentials of a combination of first-order stimulus features such as luminance, colour and edge density (see text).

Section 2 - Structured free play

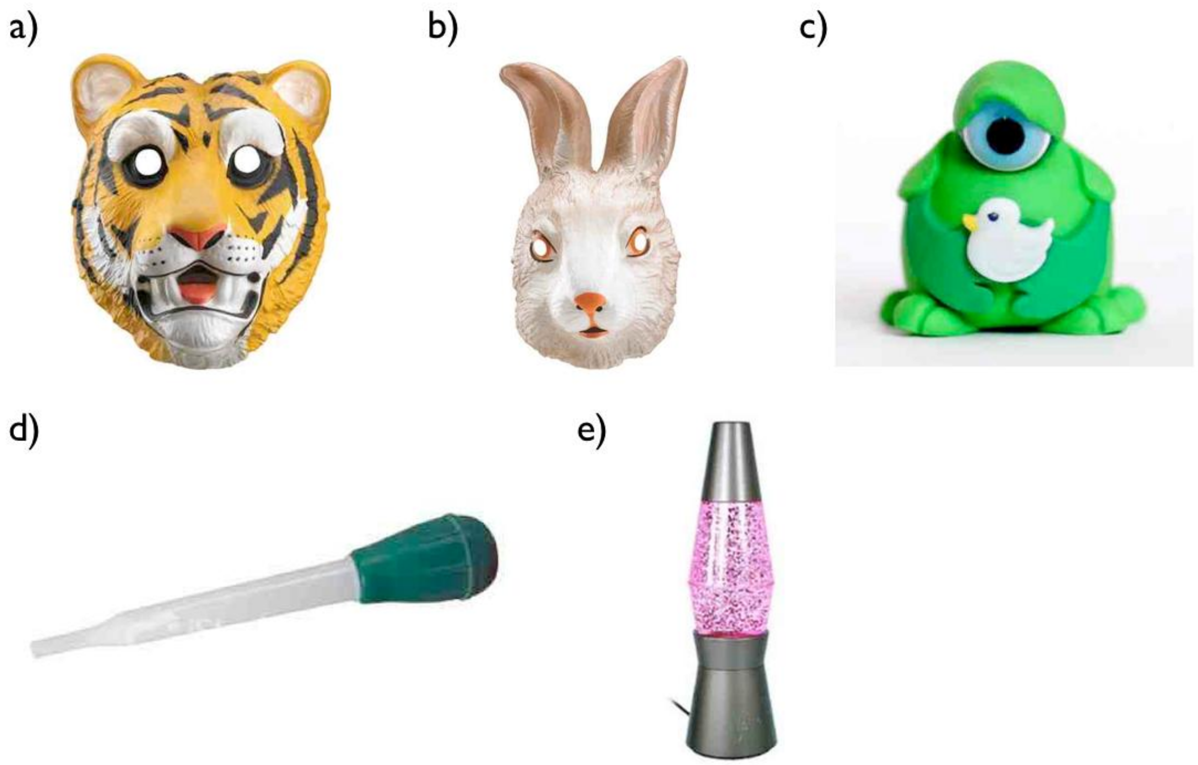

Figure S3: Stimuli used in the free play tasks. Stimulus c) is similar but not identical to that used. a) lion mask, c.25cm x 20cm. b) rabbit mask, c.30cm x 20cm. c) plastic figure, c. 10cm x 8cm. d) basting pipette, c. 25cm x 5cm. e) glitter lamp, c. 15cm x 5cm.
